# Optimization of the dilute maleic acid pretreatment of wheat straw

**DOI:** 10.1186/1754-6834-2-31

**Published:** 2009-12-21

**Authors:** A Maarten J Kootstra, Hendrik H Beeftink, Elinor L Scott, Johan PM Sanders

**Affiliations:** 1 Valorisation of Plant Production Chains, Wageningen University, PO Box 17, 6700 AA, Wageningen, The Netherlands; 2 Bioprocess Engineering Group, Wageningen University, PO Box 8129, 6700 EV, Wageningen, The Netherlands

## Abstract

**Background:**

In this study, the dilute maleic acid pretreatment of wheat straw is optimized, using pretreatment time, temperature and maleic acid concentration as design variables. A central composite design was applied to the experimental set up. The response factors used in this study are: (1) glucose benefits from improved enzymatic digestibility of wheat straw solids; (2) xylose benefits from the solubilization of xylan to the liquid phase during the pretreatment; (3) maleic acid replenishment costs; (4) neutralization costs of pretreated material; (5) costs due to furfural production; and (6) heating costs of the input materials. For each response factor, experimental data were fitted mathematically. After data translation to €/Mg dry straw, determining the relative contribution of each response factor, an economic optimization was calculated within the limits of the design variables.

**Results:**

When costs are disregarded, an almost complete glucan conversion to glucose can be reached (90% from solids, 7%-10% in liquid), after enzymatic hydrolysis. During the pretreatment, up to 90% of all xylan is converted to monomeric xylose. Taking cost factors into account, the optimal process conditions are: 50 min at 170°C, with 46 mM maleic acid, resulting in a yield of 65 €/Mg (megagram = metric ton) dry straw, consisting of 68 €/Mg glucose benefits (from solids: 85% of all glucan), 17 €/Mg xylose benefits (from liquid: 80% of all xylan), 17 €/Mg maleic acid costs, 2.0 €/Mg heating costs and 0.68 €/Mg NaOH costs. In all but the most severe of the studied conditions, furfural formation was so limited that associated costs are considered negligible.

**Conclusions:**

After the dilute maleic acid pretreatment and subsequent enzymatic hydrolysis, almost complete conversion of wheat straw glucan and xylan is possible. Taking maleic acid replenishment, heating, neutralization and furfural formation into account, the optimum in the dilute maleic acid pretreatment of wheat straw in this study is 65 €/Mg dry feedstock. This is reached when process conditions are: 50 min at 170°C, with a maleic acid concentration of 46 mM. Maleic acid replenishment is the most important of the studied cost factors.

## Background

Second generation bioethanol production uses relatively cheap, abundant and renewable agricultural by-products, such as corn stover, wheat straw or forestry residues. Compared to first generation bioethanol production, the use of lignocellulosic by-product streams results in less competition for high-quality edible carbohydrates between food and fuel application.

With annual wheat production in the European Union (EU) at 120 Tg (teragram = million metric tons), it is the largest single cereal crop in the EU; corn is the second largest at 53 Tg per year. Wheat production uses about 25 million hectare (ha), or 28% of the total harvested agricultural area, and wheat straw production is around 156 Tg per year. Assuming, first, that 1 to 2 Mg/ha of straw is left on the land in order to maintain soil quality and, secondly, that a 90% yield of ethanol from carbohydrate is achieved, the total potential for EU bioethanol production from wheat straw lies between 39 and 48 gigalitre (GL) per year [1-4]. This is about 25% to 30% of the 160 GL bio-ethanol needed to completely change from gasoline (145 GL/year) to E85 fuel (188 GL/year) in the EU. This means that about 29 - 35 GL of gasoline can potentially be replaced with bioethanol from EU wheat straw, when using E85 [5,6].

Lignocellulosic biomass requires pretreatment in order to disrupt the lignin-carbohydrate matrix and to facilitate enzymatic cellulose hydrolysis by improving cellulose accessibility to cellulolytic enzymes. This usually means a treatment that combines heat and a catalyst (acid or base). A common pretreatment uses dilute sulfuric acid (50-300 mM) at 100-200°C. The cost for pretreatment is significant; about 20% of the total production costs of second generation bioethanol production [7,8]. During hot acid pretreatment, some of the polysaccharides are hydrolyzed, mostly hemicellulose [7,9-13]. The resulting free sugars can degrade to furfural (from pentoses) or to 5-hydroxymethylfurfural (HMF; from hexoses) [14-16]. These compounds inhibit yeast cells and lead to decreased specific growth rates, specific ethanol production rates and ethanol yields. In addition, their production implies a loss of fermentable sugars [17-19].

Maleic acid and fumaric acid have been suggested as alternatives for sulfuric acid in the pretreatment. Both organic acids promote the hydrolysis of polysaccharides but, unlike sulfuric acid, neither promotes the degradation of free sugars to furfural and HMF. In recent work, both maleic and fumaric acid have been shown to be able to pretreat wheat straw; maleic acid somewhat outperforming fumaric acid. Using the two organic acids resulted in much smaller amounts of degradation products compared with using sulfuric acid [16,20-25].

Using organic acid in the pretreatment instead of sulfuric acid also significantly improves the quality of the by-product stream, as it may be more easily burned in co-firing installations, used as fertilizer or applied in animal feed [7,26,27].

Several authors have published on the optimization of pretreatment of straw-type lignocellulose materials [28-31], focusing exclusively, however, on a maximum sugar yield and disregarding pretreatment economics. In the present study, we optimize the dilute maleic acid pretreatment of wheat straw on a monetary basis. We focus on the analysis of the optimization of the maleic acid pretreatment alone - not as part of an integrated conversion process. This means that factors, such as capital costs, downstream processing costs and recycle costs, are not included in this study.

We study the influence of varying pretreatment time, temperature and maleic acid concentration on the following six factors of the resulting pretreatment:

1. glucose benefits from improved enzymatic digestibility of the raw material

2. xylose benefits from the solubilization of xylan during the pretreatment

3. costs from replenishment of lost maleic acid

4. costs due to neutralization of the pressed pretreated material

5. costs due to furfural formation from pentoses

6. heating costs of the input materials

The influence of these factors will be expressed in €/Mg straw. This means a weighing step is introduced that determines the relative contribution of each response factor to the resulting yield. In this manner, we can optimize the maleic acid pretreatment to yield the most value per Mg straw.

## Methods

### Experimental design and set up

Design-Expert 7.1.5 software (Stat-Ease, Inc, MN, USA) was used for the experimental design, model fitting and statistical data analysis. In order to reduce the number of experiments needed, a central composite factorial design was applied; the experimental conditions are mentioned in the 'Wheat straw pretreatment' section. Experimental data for each response factor were expressed in mathematical models. The starting point was a quadratic model which was then adjusted by backward elimination: taking out terms that had no significant contribution (*P *> 0.05) one by one, and then recalculating the model with the remaining terms.

### Preparation and analysis of wheat straw

Wheat straw (harvest September 2006, Delfzijl, The Netherlands) was milled twice; first in a Pallmann mill (4 × 30 mm sieve) and then in a Retsch mill (1 mm sieve). Milled straw was kept in a sealed plastic barrel at room temperature until used. Chemical composition was analyzed in triplicate, as described by TAPPI methods [32-37], with minor modifications: (1) samples were extracted with ethanol:toluene 2:1, 96% (v/v) ethanol and hot water (1 h) at boiling temperature; (2) the extracted samples were dried at 60°C for 16 h; (3) monomeric sugar and lignin content of the ethanol-extracted material was determined after a two-step hydrolysis with sulfuric acid (12 M for 1 h at 30°C; 1 M for 3 h at 100°C); (4) acid soluble lignin in the hydrolyzate was determined by spectrophotometric determination at 205 nm.

Monomeric sugars were measured by High Performance Anion Exchange Chromatography with Pulsed Amperometric Detection (HPAEC-PAD). A Dionex system with Carbopak PA1 column with pre-column was used at 30°C, with de-ionized water as the mobile phase (1 mL/min) and fucose as the internal standard. The Dionex high-performance liquid chromatography (HPLC) method was also used for the determination of monomeric sugars in the aqueous phase of both pretreated and enzymatically hydrolyzed wheat straw. Dry matter content was 91.8% (w/w) (24 h at 105°C). The chemical composition of the used wheat straw is shown in Table 1.

**Table 1:** Chemical composition (dry-weight basis) of the wheat straw used in this study.

Component	Content (% w/w)
Glucan	36.3
Xylan	19.0
Arabinan	2.1
Galactan, mannan, rhamnan	< 0.6 each
Uronic acids	2.1
Lignin	25.5
Extractives	7.8
Protein	3.3
Ash	6.7

### Wheat straw pretreatment

All chemicals were of research grade and used as received (maleic acid: Aldrich M153). Milled wheat straw (8.0 g; 7.34 g dry matter) was mixed in poly-ethylene containers with 65.5 mL of maleic acid solution (11, 50 or 89 mM), resulting in 10% (w/w) dry straw solids loading. The straw/acid mixture was soaked for 20-24 h at room temperature and then transferred to 316L stainless steel reactors (inner height × diameter: 90.0 × 40.0 mm; 5.0 mm wall), fitted with thermocouples. Reactors were heated in a Haake B bath with a Haake N3 temperature controller (Thermo Fisher Scientific, MA, USA), filled with silicon oil (DC 200 fluid, 100 cSt, Dow Corning, MI, USA). Sample core temperature was recorded (Picotech data collector and software; Picotech, Cambridgeshire, UK). Pretreatments were performed at 130°C, 150°C and 170°C. Holding time was 10, 30 and 50 min, starting from when the desired core temperature was reached. Heating bath oil was preheated to 1°C above the desired the temperature. During the holding time, the temperature inside the reactors never differed more than 1°C from the desired temperature. After the reaction time, the reactors were cooled by quenching in ice water.

### Solid-liquid separation: press step

The pretreated material was pressed in a custom built hydraulic press. The inner diameter of the press was 40.0 mm and the free moving speed was 10 mm/s. Press time was 10 s, starting from when maximum pressure of 200 bar was reached. A filter was placed (0.5 mm thick, 39.0 mm diameter; +/- 400 holes of 0.8 mm diameter, evenly distributed) on the porous bottom of the press. Both press and filter were made of 316L stainless steel. Pressed pellets and pressed out aqueous phase were collected and stored at -20°C until, respectively, enzymatic hydrolysis and analysis.

### Enzymatic hydrolysis

After the pretreatment and press step, the resulting pellets were dried (48 h at 60°C, under vacuum) and transferred to 250 mL baffled shake flasks. De-ionized water was added to dilute to 5% (w/w), based on original straw dry weight, taking into account water added with the Na-azide solution (0.05% [w/w] final concentration to prevent microbial growth), during pH adjustment to 5.0 with 0.1 and 1 M NaOH solution and with the enzyme addition. At the start of the enzymatic hydrolysis, 0.4 mL per g dry matter straw of GC220 cellulase enzyme mixture was added (batch 4900759148, 7608 IU/mL cellulase activity, Genencor, NY, USA), corresponding to 46 FPU/g original dry matter straw [38]. This relatively high dosage is in the plateau region of the dose-effect curve of the enzyme mixture. This was to ensure the effect of the pretreatment on the glucose yield would be measured, not the effect of the enzyme concentration.

Flasks were left overnight for the pH to equilibrate. After pH fine tuning and enzyme addition, flasks were closed with airtight plugs and placed in an Innova 44 incubator shaker (50°C, 150 rpm, 2 inch stroke; NBSC, NJ, USA). Samples of 1.5 mL were taken at *t *= 0 and 96 h; after 5 min enzyme inactivation at 90°C, samples were stored at -20°C until analysis.

The glucose yield from cellulose was calculated as follows:(1)

where GS is the amount of glucose present in the sample of dry straw (g glucose equivalents in cellulose) and GH is the amount of glucose (g) present in the aqueous phase of the hydrolyzate, after enzymatic hydrolysis of the pellet. Xylose yield was calculated similarly, using xylan/xylose content. The fact that the form in which xylose is stored in wheat straw hemicellulose is arabinoxylan rather than xylan was ignored.

### Organic acid and sugar degradation product analysis

Maleic acid, fumaric acid, furfural, and 5-HMF concentrations after pretreatment were measured by HPLC. Measurements were performed in the liquid phase prior to starting the enzymatic treatment. A Waters system with Shodex Ionpak KC-811 column at 30°C with a Fast Fruit Juice Guard-Pak pre-column was used. Mobile phase (1 mL/min) was 3.65 mM phosphoric acid, internal standard was phenoxyacetic acid and peak detection was done with ultraviolet (210/280 nm).

### Analysis of oligomeric sugars

To assess the amount of oligomeric sugars present in the liquid phase after the pretreatment, a method closely resembling National Renewable Energy Laboratory (NREL) LAP-014 [39] was used. One millilitre samples were used for a secondary hydrolysis, in triplicate. This was performed using 4% (w/w) H_2_SO_4 _at 121°C during 10 min. Monomeric sugars as well as degradation products were measured, as described above, and, comparing these results with the values from just after the pretreatment, the original amounts of oligomeric sugars were calculated, taking into account the extra formation of degradation products during the secondary hydrolysis.

### Titration of liquid phase

The titration of the liquid phase was performed using a Metrohm 718 stat titrino set (Metrohm, Herisau, Switzerland), with the titration vessel equipped with a thermostatic jacket (25°C). Of each sample, 3 mL of the liquid phase was brought to pH 5.0 with 50 mM NaOH. Analyses were performed in duplicate.

### Optimization of benefits and cost factors

The costs and benefit factors used in this study are defined as follows:

(1) Glucose benefits: the benefits from glucose that is released from the pressed pretreated material, after enzymatic hydrolysis. This shows the effect of the pretreatment on (increasing) the enzymatic digestibility of the raw material.

(2) Xylose benefits: the benefits from the liquid phase, after the solid-liquid separation. Both oligomeric and monomeric xylose that are solubilized during the pretreatment are taken into account.

(3) Costs for the maleic acid that is not recovered in the liquid phase after the solid-liquid separation and, therefore, needs to be replenished.

(4) Costs for NaOH needed to neutralize the pressed pretreated material prior to the enzymatic hydrolysis. This in effect combines two sub-factors: (a) the amount of NaOH needed per volume of liquid phase to set the pH to 5 and (b) the total amount of this liquid phase still present in the pressed pellet, or in other words: the efficiency of the solid-liquid separation. These two sub-factors may depend differently on the process conditions.

(5) Costs that arise due to furfural formation from pentoses.

(6) Heating costs of the straw that enters the process and of the water that needs to be replenished.

All data is expressed in €/Mg straw, a weighing step that reveals the relative contribution of each factor. The total benefits (that is the economic yield of the pretreatment) was calculated by subtracting all costs from the glucose benefits. Within the limits of the design space, an optimization was calculated.

## Results and discussion

### Effect of pretreatment conditions on glucose benefits

The main goal of the pretreatment of lignocellulosic material is to increase the enzymatic digestibility. In order to study the influence of pretreatment time, temperature and maleic acid concentration on the pretreatment of wheat straw, the enzymatic digestibility of the solid pretreated material was determined, after solid-liquid separation. The resulting glucose yield, as determined after subsequent enzymatic hydrolysis, was translated to €/Mg straw glucose benefits (with glucose at 200 € per Mg [40]). The fact that a more concentrated glucose stream has a higher value per amount of glucose than a less concentrated stream is ignored.

**Figure 1:**
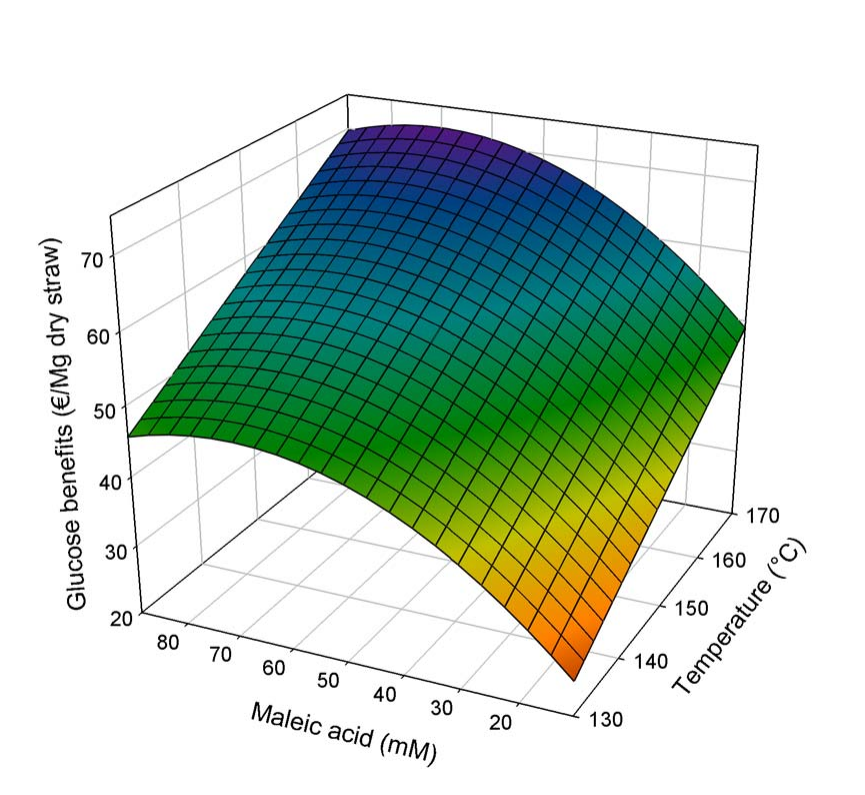
**Three-dimensional response surface for glucose benefits (€/Mg dry straw) in relation to pretreatment conditions**. Pretreatment time set at 50 min.

The model for the experimental results is based on the significant effects of all three factors, extended with the squared factor of maleic acid concentration (see Equation 2). The quadratic model is significant (*P *< 0.0001) and fits the experimental data with *R*^2 ^_adjusted _= 0.94 (see Additional file 1). In Figure 1, details of the response analysis of the results on glucose yield from the solid phase are shown as a three-dimensional surface.(2)

with Bene_glu _= glucose benefits (€/Mg dry straw), *A *= (*t*-*t*_C_)/*t*_S_; *B *= (*T*-*T*_C_)/*T*_S_; *C *= (*M*_A_-*M*_A, C_)/*M*_A, S_; *t *= pretreatment time (min), *T *= pretreatment temperature (°C) and *M*_A _= concentration maleic acid (mM); subscript C = centre value, subscript S = step value; *t*_C _= 30 min; *t*_S _= 20 min; *T*_C _= 150°C; *T*_S _= 20°C; *M*_A, C _= 50 mM; *M*_A, S _= 39 mM.

As shown in Equation 2 and Figure 1, within the studied design space, increasing the digestibility (expressed as glucose yield after subsequent enzymatic hydrolysis) is strongly dependent on increasing the pretreatment temperature. Increasing pretreatment time from 10 to 50 min also has a positive effect, but less so. The influence of the maleic acid concentration has a negative quadratic part, resulting in a maximum enzymatic glucose yield at 70.4 mM maleic acid (50 min at 170°C). On the basis of the fit to the data, a maximum glucose yield of 71.87 €/Mg of straw is predicted, representing 90% of all glucan present in the original straw.

During the pretreatment, high concentrations of maleic acid seem to favor the formation of monomeric glucose in the liquid phase (Table 2). Since most of the liquid phase is pressed out, raising the maleic acid concentration in the pretreatment will eventually result in a negative effect on the enzymatic glucose yield from the pressed material.

**Table 2:** Glucose mass balance of maleic acid pretreatment of wheat straw.

Run	Pre-treatment time (min)	Pre-treatment temperature (°C)	Maleic acid concentration (mM)	Glucose monomers in aqueous (%)	HMF in aqueous (%)	Glucose oligomers in aqueous (%)	Glucose in pressed material (%) Not corrected for a, b, and c	Glucose in pressed material (%) Corrected for a, b, and c	Total (%)
				**a**	**b**	**c**	**d**	**e**	**a+b+c+e**

1	10	130	11	1	0	2	24	24	27
2	50	130	89	2	0	5	54	54	61
5	50	170	11	1	0	6	63	63	70
6	10	170	89	8	1	1	79	78	88
7	10	170	11	1	0	5	46	46	52
8	50	170	89	10	1	0	82	81	92
9	10	130	89	1	0	6	45	45	52
11	50	130	11	1	0	4	36	36	41
14	30	130	50	1	0	6	44	44	50
15	50	150	50	4	0	4	71	71	79
16	10	150	50	2	0	6	61	61	69
17	30	150	89	6	0	2	72	72	81
18	30	150	11	1	0	4	36	36	41
19	30	170	50	7	1	1	82	81	91
c×6	30	150	50	3 (0.1)	0 (0.0)	5 (0.2)	66 (1.0)	66 (1.0)	74 (0.9)

c×6 = centre runs, six repeats. Average value given. Standard deviation between brackets. Percentages are expressed as glucose equivalents, as a fraction of total glucose equivalents present in untreated wheat straw.

Within the experimental results, the maximum glucose yield from the pressed material was 82% of the total of glucose present in the original straw. Under these conditions (50 min, 170°C, and 89 mM maleic acid), 10% of the total glucose from the straw was detected as monomeric sugar in the liquid phase. Only 1% of the total glucose had been degraded to HMF and only traces of oligo-glucans were present in the liquid phase, closing the glucose mass balance for 92% (see Table 2).

The glucose yields in this study are somewhat lower than in a previous work with maleic acid pretreatment of wheat straw, where 96% of all glucan was retrieved as monomeric glucose after enzymatic hydrolysis [25]. However, the resulting maximum glucose yield of the present study (combining liquid and solid fraction to 92% of total present glucose) is in line with other studies on pretreatment of straw-like lignocellulosic materials [28-31]. In fact, the model predicts a maximum glucose yield from the pretreated solids of 90% of all glucan present in straw, while Table 2 implies that between 7% to 10% of all glucose would be present in the liquid phase. This suggests that 97% to 100% conversion of glucan to glucose can be reached, using maleic acid in the pretreatment. However, it also means that, in order to use more than 90% of all glucose, the glucose that is solubilized in the liquid phase would need to be included.

### Effect of pretreatment conditions on xylose benefits

During the pretreatment, a large part of the hemicellulose is solubilized. When the resulting xylose monomers and oligomers are taken into account for the optimization, the total benefits per Mg straw can be raised. For this study, we estimated the value of xylose at 100 €/Mg, 50% of the value of glucose [40]. As with glucose, the fact that a more concentrated glucose stream has a higher value per amount of xylose than a less concentrated stream is ignored. In Table 3, the xylose mass balance is shown, clarifying that xylans are solubilized as oligomeric and monomeric xylose. For this study, both types are included as equally valuable in the xylose benefits calculation, ignoring the costs for an additional hydrolysis that might be needed to convert remaining oligomeric xylose to monomers.

**Table 3:** Xylose mass balance of maleic acid pretreatment of wheat straw.

Run	Pre-treatment time (min)	Pre-treatment temperature (°C)	Maleic acid concentration (mM)	Xylose monomers in aqueous (%)	Furfural in aqueous (%)	Xylose oligomers in aqueous (%)	**Xylose in pressed material (%)**. Not corrected for a, b, and c	**Xylose in pressed material (%)**. Corrected for a, b, and c	Total (%)
				**a**	**b**	**c**	**d**	**e**	**a+b+c+e**

1	10	130	11	0	0	2	18	18	20
2	50	130	89	44	1	22	25	19	86
5	50	170	11	5	3	47	31	26	81
6	10	170	89	85	7	1	15	6	100
7	10	170	11	1	1	26	37	34	61
8	50	170	89	72	18	1	11	4	94
9	10	130	89	15	0	35	26	21	72
11	50	130	11	1	0	11	31	30	42
14	30	130	50	7	0	35	27	23	65
15	50	150	50	66	3	11	24	16	96
16	10	150	50	36	1	32	26	19	87
17	30	150	89	78	4	5	20	12	99
18	30	150	11	2	0	11	32	30	43
19	30	170	50	75	10	4	17	9	98
c×6	30	150	50	58 (2.2)	2 (0.1)	17 (1.0)	24 (0.3)	17 (0.2)	94 (1.4)

c×6 = centre runs, six repeats. Average value given. Standard deviation between brackets. Percentages are expressed as xylose equivalents, as a fraction of total xylose equivalents present in untreated wheat straw.

**Figure 2:**
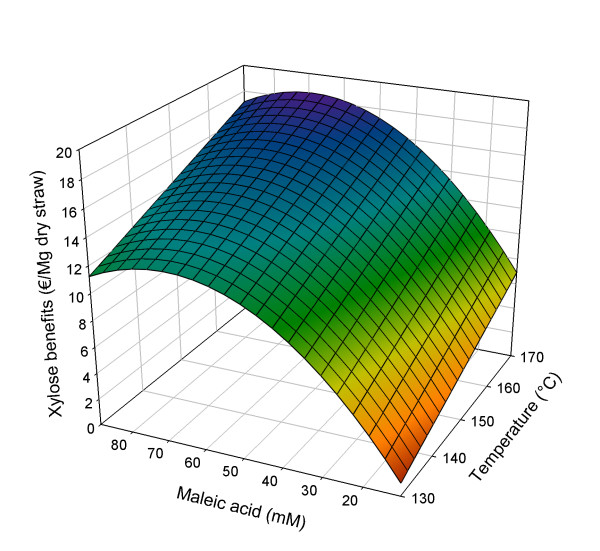
**Three-dimensional response surface for xylose benefits (€/Mg dry straw) in relation to pretreatment conditions**. Pretreatment time set at 50 min.

The model for the experimental results is based on the significant effects of the pretreatment temperature and maleic acid concentration, extended with the squared factor of the latter (see Equation 3). The quadratic model is significant (*P *< 0.0001) and fits the experimental data with *R*^2^_adjusted _= 0.90 (see Additional file 1). In Figure 2, details of the response analysis of the results on glucose yield from the solid phase are shown as a three-dimensional surface.(3)

with Bene_xyl _= xylose benefits (€/Mg dry straw), *B *= (*T*-*T*_C_)/*T*_S_; *C *= (*M*_A_-*M*_A, C_)/*M*_A, S_; *T *= pretreatment temperature (°C) and *M*_A _= concentration maleic acid (mM); subscript C = centre value, subscript S = step value; *T*_C _= 150°C; *T*_S _= 20°C; *M*_A, C _= 50 mM; *M*_A, S _= 39 mM.

As shown in Equation 3 and Figure 2, within the studied design space, increasing the xylose yield is strongly dependent on increasing the maleic acid concentration, as well as the pretreatment temperature. Increasing pretreatment time from 10 to 50 min does not have a significant influence on the xylose yield. The influence of the maleic acid concentration has a negative quadratic part, resulting in a maximum xylose yield from the pretreated liquid phase at 68.9 mM maleic acid and 170°C. Under these conditions, the model predicts a maximum xylose yield of 18.55 €/Mg of straw, representing 85% of all xylan present in the original straw. The xylose that is solubilized under these conditions will largely be monomeric (Table 3) and about 5% of all solubilized xylose remains in the liquid phase of the pressed pellet. It is clear that, in the maleic acid pretreatment, almost complete xylan conversion to xylose is quite possible but a part of this xylose will degrade to furfural. Obviously, the idea is to minimize loss of xylose to furfural formation.

Within the experimental results, the maximum xylose yield from the liquid phase was 81% of the total of xylose present in the original straw. That is, 86% of all xylan was solubilized but, as not all liquid phase is pressed out, some solubilized xylose remains in the liquid phase of the pressed material. Under these conditions (10 min, 170°C, and 89 mM maleic acid), 85% of the total xylose from the straw was detected as monomeric sugar in the liquid phase. Only 1% of the total xylose was present in oligomeric form, 7% of all xylose had had been degraded to furfural and 6% remained as polysaccharide in the pressed solid phase, closing the xylose mass balance for 100% (Table 3). The xylose yields in this study are very comparable with previous work with maleic acid pretreatment of wheat straw and corn stover [21,25].

### Effect of pretreatment conditions on maleic acid costs

After the pretreatment, not all of the maleic acid is detected in the liquid phase that is pressed out during the solid-liquid separation. Some of the maleic acid is degraded, while another part continues downstream with the pressed solid phase. When recycling the maleic acid for use in further pretreatments of fresh straw, both these fractions of acid are considered lost and need to be replenished. The experimental results of this study represent the cost of all maleic acid that is lost, expressed as €/Mg straw, with maleic acid at 1000 €/Mg [41]. For this study, it is assumed that all maleic acid that is detected can be recycled at no additional cost. Only the acid that needs to be replenished is considered.

**Figure 3:**
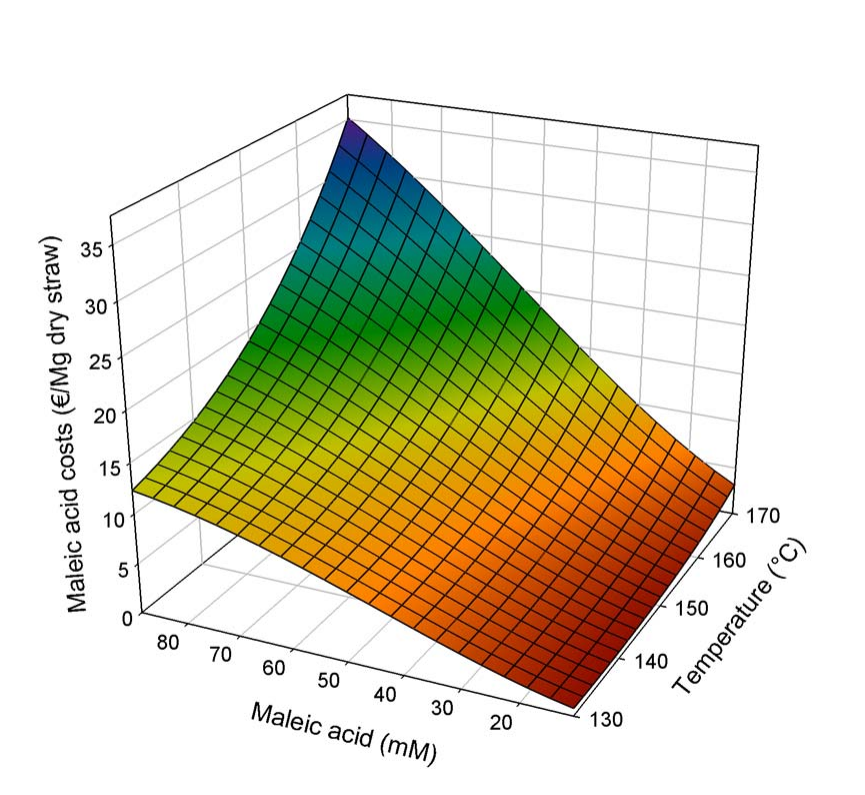
**Three-dimensional response surface for maleic acid costs (€/Mg dry straw) in relation to pretreatment conditions**. Pretreatment time set at 50 min.

The model used to describe the experimental results is based on the effects of all three factors, extended with parameters for interactions and squared factors (see Equation 4). A square root transformation of the response factor was applied for improved model fit. The quadratic model is significant (*P *< 0.0001) and fits the data with *R*^2^_adjusted _= 1.00 (see Additional file 1). In Figure 3, details of the response analysis of the results on acid loss from the liquid phase are shown as a three-dimensional surface.(4)

with Costs_MA _= maleic acid costs (€/Mg dry straw), *A *= (*t*-*t*_C_)/*t*_S_; *B *= (*T*-*T*_C_)/*T*_S_; *C *= (*M*_A_-*M*_A, C_)/*M*_A, S_; *t *= pretreatment time (min), *T *= pretreatment temperature (°C) and *M*_A _= concentration maleic acid (mM); subscript C = centre value, subscript S = step value; *t*_C _= 30 min; *t*_S _= 20 min; *T*_C _= 150°C; *T*_S _= 20°C; M_A, C _= 50 mM; *M*_A, S _= 39 mM.

As can be seen in Equation 4 and Figure 3, minimizing costs due to acid loss within the studied design space is, for a large part, dependent on concentration of maleic acid that is present at the start of the pretreatment. As may be expected, the lower the maleic acid concentration, the lower the costs for replenishing any acid that is lost. However, increasing acid concentration results in higher costs for acid loss (results not shown). This may be not only because a higher concentration is left in the pellet after pressing but also because the isomerization to fumaric acid itself is described as acid catalyzed [42,43].

Minimal acid loss within the studied design space is 0.54 €/Mg straw and occurs at a 10 min pretreatment with 11 mM maleic acid at 152°C. In comparison, maximal acid costs occur when the pretreatment is most severe in this study: 50 min at 170°C, with 89 mM maleic acid, results in a staggering 34.88 €/Mg straw.

It is striking that at shorter, less acidic pretreatments, there appears to be a small drop in acid loss when the temperature is raised from 130° to around 150°C (max drop is about 0.95 €/Mg straw). This may be explained by an increase of the amount of liquid that is pressed out (results not shown), while the duration of the pretreatment was not long enough to allow for extensive maleic acid degradation (mostly isomerization into fumaric acid) during the time of the pretreatment [44,45]. When pretreatments are performed with more acid for longer time, more acid is degraded, no longer resulting in this reduction of acid costs around 150°C.

Furthermore, for minimizing acid costs, high pretreatment temperatures are best avoided (but the influence of increasing the temperature is less than increasing the acid concentration). Increasing pretreatment time only seems to have minimal influence.

### Effect of pretreatment conditions on sodium hydroxide costs

After the solid-liquid separation, the amount of NaOH needed to set the pH of the solid phase depended on two sub-factors: the amount of NaOH needed per volume of liquid to set the pH to 5 (comparable to 'acid number') and the volume of liquid remaining in the solid phase. In this study, both sub-factors reacted oppositely to changes in pretreatment time and temperature (results not shown), meaning that, as the solid-liquid separation gets somewhat more efficient when pretreatment conditions change, the same changes cause the remaining liquid phase to need more NaOH per volume to set the pH to 5. This may be due to acetic and uronic acids being released from the straw during the pretreatment (results not shown). In short, the changes in the two sub-factors due to changes in pretreatment time and temperature cancel each other out. This leads to the amount of NaOH needed to set the pressed pretreatment solids to pH 5 only being significantly dependent on the maleic acid concentration of the pretreatment. The model used to describe the experimental results as NaOH cost per Mg of straw (with NaOH costing 575 €/Mg [46]) is therefore linear, as can be seen in Equation 5. The model is significant (*P *< 0.0001) and fits the data with *R*^2 ^_adjusted _= 0.98. The costs for NaOH within the studied design space vary between 0.15 and 1.81 €/Mg dry straw, for when 11 and 89 mM maleic acid was used, respectively.(5)

with Costs_NaOH _= NaOH costs (€/Mg dry straw), *C *= (*M*_A_-*M*_A, C_)/*M*_A, S_; *M*_A _= concentration maleic acid (mM); subscript C = centre value, subscript S = step value; *M*_A, C _= 50 mM; *M*_A, S _= 39 mM

### Effect of pretreatment conditions of furfural production

The formation of furfural during the maleic acid pretreatment is very limited, confirming results of earlier studies [21,25,47]. Under most pretreatment conditions, only minor amounts of furfural are formed. In only one of the experiments (50 min, 170°C, 89 mM acid), slightly more than 3 g/L of furfural was formed. This is close to 30 mM at which furfural and HMF have been reported to be inhibitory to yeast in the production of ethanol from glucose, although adaptation of yeast to similar concentrations has also been reported [48-50].

The model used to describe the experimental results is based on the effects of all three factors, extended with the interaction of temperature and acid concentration and the squared factor of the latter (see Equation 6). A logarithmic transformation of the response factor was applied for improved model fit. The quadratic model is significant (*P *< 0.0001) and fits the data with *R*^2^_adjusted _= 0.94 (see Additional file 1).(6)

with Fur = furfural concentration (g/L), *A *= (*t*-*t*_C_)/*t*_S_; *B *= (*T*-*T*_C_)/*T*_S_; *C *= (*M*_A_-*M*_A, C_)/*M*_A, S_; *t *= pretreatment time (min), *T *= pretreatment temperature (°C) and *M*_A _= concentration maleic acid (mM); subscript C = centre value, subscript S = step value; *t*_C _= 30 min; *t*_S _= 20 min; *T*_C _= 150°C; *T*_S _= 20°C; *M*_A, C _= 50 mM; *M*_A, S _= 39 mM.

**Figure 4:**
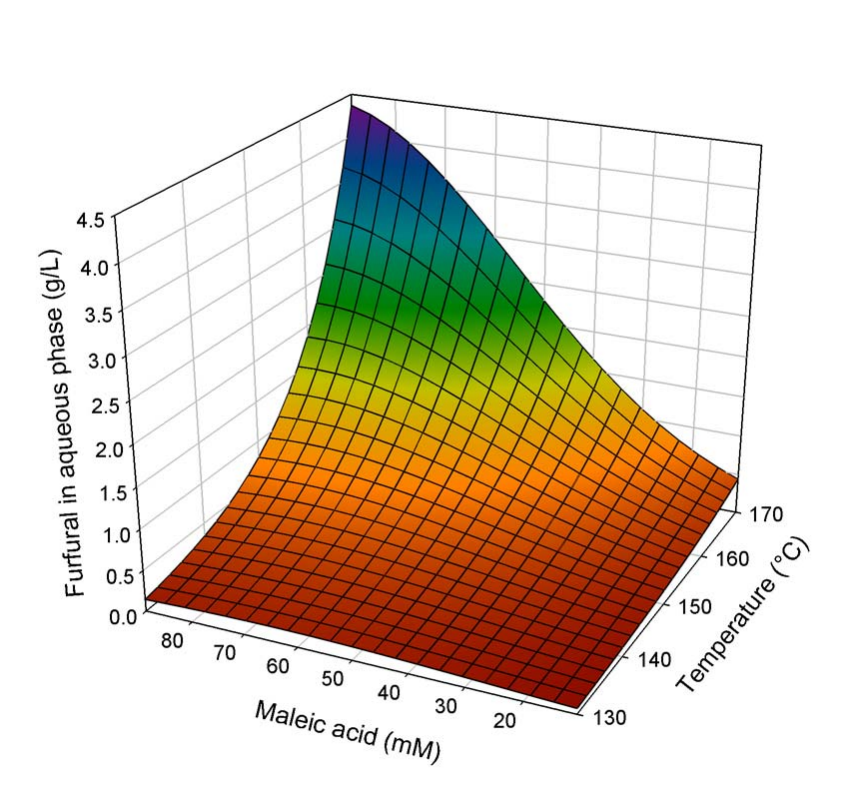
**Three-dimensional response surface for furfural formation (g/L in liquid phase) in relation to pretreatment conditions**. Pretreatment time set at 50 min.

In Figure 4, details of the response analysis of the results on furfural formation in the liquid phase are shown as a three-dimensional surface. The furfural concentration is depicted on a linear scale, emphasizing, together with Equation 6, the very low furfural formation under most conditions, except the most extreme. The negative quadratic term in the equation somewhat lessens the influence of increasing the acid concentration.

Taking into account the experimental results, it can be assumed that the influence of furfural formation on the economics of the studied maleic acid pretreatment is zero, provided that the most extreme conditions are avoided. The furfural concentrations that are formed are usually low enough to avoid inhibition of yeast in the bio-ethanol production [48-52].

However, even though very limited in this study, furfural formation should not be completely disregarded. First, because production of furfural means loss of xylose, leading to a lower potential xylose yield and lower associated benefits. Secondly, if the liquid phase is reused for pretreatment of fresh material, build up of furfural could occur, possibly still resulting in non-negligible inhibitory furfural concentrations, and in higher associated process costs.

### Effect of pretreatment conditions on heating costs

Heating lignocellulose means heating costs and heating wheat straw to 170°C costs more than to 130°C. Heating costs that are taken into account are those for the straw entering the pretreatment process, and also for the water that needs to be replenished, as some of it leaves the pretreatment process after the solid-liquid separation, as part of the solid phase that continues to the enzymatic hydrolysis. As, in this study, the solid phase after solid-liquid separation usually consisted of around 40% dry matter, with pretreatment time and temperature only having a negligible influence (results not shown), it is assumed that for every Mg of dry straw that enters the pretreatment 1.5 Mg of water needs to be included and it has to be heated as well. Considering the relatively small specific surface area of large scale process equipment, it is assumed that only negligible amounts of heating energy are needed during the holding time of the pretreatment, the solid-liquid separation and the recycle of water and acid. Using a calculated specific heat capacity of dry straw of 1.7 Jg^-1^K^-1 ^and energy costs from coal values [53,54], the heating costs for the process can be described with Equation 7.(7)

with Costs_heating _= heating costs (€/Mg dry straw), *T *= pretreatment temperature (°C); subscript C = centre value, subscript S = step value; *T*_C _= 150°C; *T*_S _= 20°C.

In short, the heating cost of the maleic acid pretreatment in the studied design space vary from 1.51 to 2.06 €/Mg dry straw, for 130° to 170°C, respectively.

### Optimal economic yield

**Figure 5:**
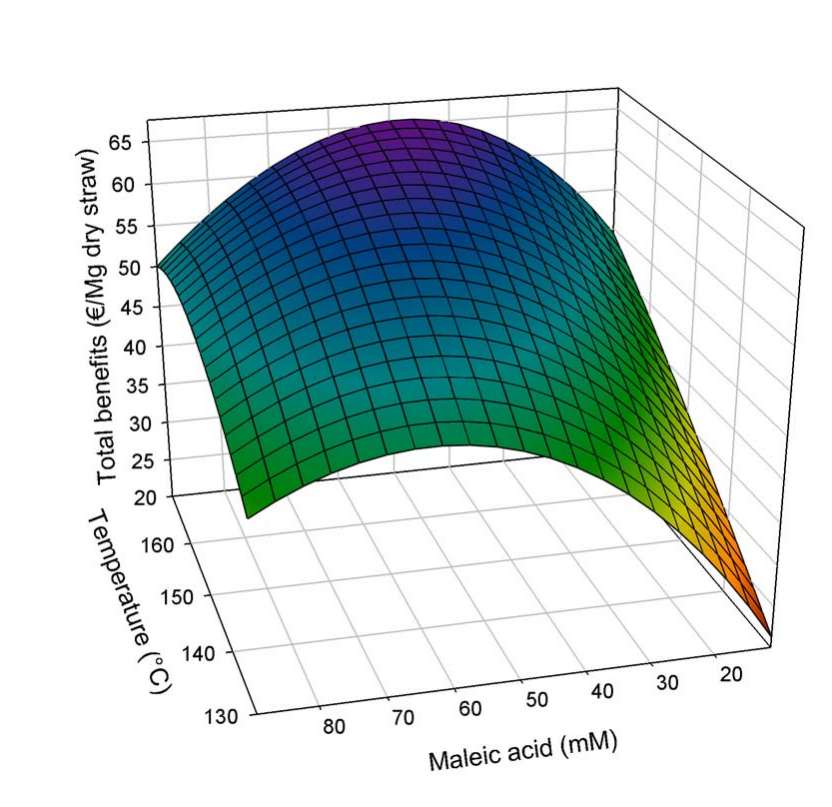
**Optimization of total benefits (€/Mg dry straw), including xylose, in relation to pretreatment conditions**. Pretreatment time set at 50 min.

Taking the benefits from glucose and xylose and subtracting the costs of maleic acid, heating and NaOH, results in an economic optimum of the maleic acid pretreatment at a reaction time of 50 min, at 170°C, in presence of 46.21 mM maleic acid (see Figure 5). This would mean a glucose yield of 68.03 €/Mg dry straw (85% of all glucan), a xylose yield of 16.74 €/Mg dry straw (close to 80% of all xylan), maleic acid costs of 16.55 €/Mg dry straw, heating costs of 2.06 €/Mg straw and NaOH costs of 0.90 €/Mg straw. The total resulting economic yield at this optimum would then be 65.26 €/Mg straw. A pretreatment time of 10 or 30 min (results not shown) leads to a lower optimal economic yield (64.34 and 64.90 €/Mg straw, respectively), at 170°C and at higher acid concentrations (53.07 and 49.72 mM, respectively). Pretreatment time does have an influence on the optimal economic yield, but less so than pretreatment temperature and maleic acid concentration.

**Figure 6:**
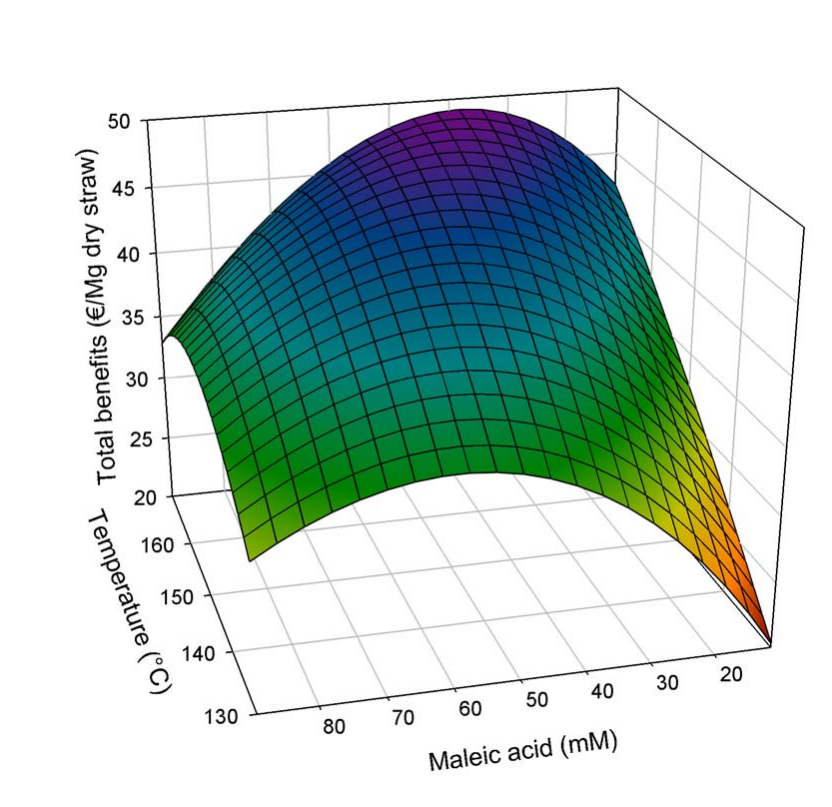
**Optimization of total benefits (€/Mg dry straw), not including xylose, in relation to pretreatment conditions**. Pretreatment time set at 50 min.

Since the xylose benefits are somewhat speculative, and the contribution of glucose to the total benefits is about three to four times larger than that of xylose, an optimization using only glucose benefits but still all cost factors is also performed (see Figure 6).

The reasons for the xylose yield being somewhat speculative are: first, the estimated xylose value of half of that for glucose is quite uncertain; secondly, the fit of the xylose data is not as good as that for the glucose data; and, thirdly, in the case of glucose, the fermentable sugars are released during enzymatic hydrolysis and can relatively easily be transformed to ethanol by fermentation. For xylose, the solubilization takes place at lower concentration in water that contains all acids and many other constituents. This implies a complication of the application of xylose and, therefore, adds uncertainty to its value.

Using only the benefits from glucose, the optimal conditions are again at a reaction time of 50 min, at 170°C, but now in presence of 36.2 mM maleic acid (see Figure 6). As the optimal conditions for both glucose and xylose yields are similar, it is as expected that not taking xylose benefits into account would result in an economic optimum at lower maleic acid concentration.

We therefore reach a glucose yield of 64.11 €/Mg dry straw (corresponding to 80% of all glucan present in straw), maleic acid costs of 12.01 €/Mg dry straw, heating costs of 2.06 €/Mg straw and NaOH costs of 0.68 €/Mg straw. The total resulting economic yield at this optimum would be 49.35 €/Mg straw. Both the maleic acid concentrations of 36.2 and 46.2 mM would result in furfural concentration of 2 g/L or lower, which confirms the assumption of zero costs associated with furfural formation.

Logically, the glucose (and xylose) yields, when using the optimal economic pretreatment, are somewhat lower than maximally could be achieved. As a result of the rapidly increasing acid costs, it is more economical to settle for a less acidic pretreatment that yields a lower-than-maximum amount of glucose (and xylose). If 90% of all glucans in the straw were yielded as glucose from the pretreatment solids, another 6.4 € per Mg straw could be achieved. However, the acid costs would rise with an additional 15 € to 27 €/Mg straw (and the NaOH costs with 0.72 to 1.41 €/Mg straw).

A total yield of around 49 €/Mg of dry straw, or 65 €/Mg when taking xylose benefits into account, is fairly promising as theoretical sugar benefits are around 80 and 20 €/Mg dry straw for glucose and xylose, respectively. When taking into account feedstock costs of 25 to 33 €/Mg (corn stover [8,55]), it is clear that process economics are somewhat challenging.

In a comparable dilute sulfuric acid process, in which both glucose and xylose are assumed to be of similar value for ethanol production, the projected total ethanol production costs are 320 €/Mg ethanol. Enzyme costs are estimated at 24 €/Mg ethanol or 6 €/Mg feedstock. The pretreatment costs account for around 16 €/Mg feedstock, more than half of which (around 9 €/Mg feedstock) is due to the high capital cost of the pretreatment reactors that need to deal with the corrosive sulfuric acid [8]. Since the organic maleic acid is less corrosive, a lower capital cost for the pretreatment can be expected. Processing equipment, such as vessels and piping made of the more corrosion resistant 316L steel, can be 20% to 50% more expensive than when made of standard 304 steel [56]. Another matter that should be taken into account is the possibility that, when using sulfuric acid, extra features such as corrosion resistant storage vessels or more stringent safety and handling measures, compared to the application of the weaker (and solid) maleic acid may be needed. We estimate the extent of this difference to be significant but this, of course, greatly depends on the process conditions of the maleic acid pretreatment. On the other hand, maleic acid is more expensive than sulfuric acid but the much reduced sugar degradation and the organic by-product stream from maleic acid offer advantages over the sulfuric acid treatment. Clearly, more study is needed in order to compare the economics of these two pretreatments.

Several options exist in order to raise the economic yield of the maleic acid pretreatment. First, the sugar yield could be somewhat raised by increasing the severity of the pretreatment [57]. However, as shown earlier, this should be done without raising the maleic acid concentration or the pretreatment temperature (to a higher value than in the studied design space), since both would quickly increase acid costs (also a higher temperature would increase heating costs and a higher maleic acid concentration would increase NaOH costs). This leaves only the pretreatment time to be increased to outside the studied design space, as this is the process variable which least affects maleic acid costs (in the studied design space). Heating costs would not increase, assuming that maintaining a certain temperature for longer requires much less energy than heating to a higher temperature. However, it should not be overlooked that increasing the process time will lead to higher process costs, since process throughput will be reduced, decreasing plant output per unit of capital investment.

A second option is to lower acid costs. For example, it may not be necessary to replenish all of the maleic acid that is lost in the pretreatment. The fumaric acid that results from the isomerization of maleic acid during the pretreatment also possesses pretreatment potential, albeit less than maleic acid [25,47]. In this study, up to 12% of maleic acid is transformed to fumaric acid (results not shown). It may, therefore, be possible to save a significant part of the maleic acid costs in this manner. However, applying this strategy in the recycle and re-use of the acids in a (semi-) continuous process implies a mixed fumaric-maleic acid pretreatment. It is not known to what extent the pretreatment efficiency of fumaric acid that is formed can simply be added to that of the maleic acid which is already present. This remains to be studied further, as it falls outside of the scope of the current study.

Another way to lower acid costs would be to use an acid that is cheaper per Mg pretreated straw and/or to make acid *in situ*. For example, the xylose that is released in the pretreatment may be converted to lactic acid [58], which may be used in the pretreatment (although it is weaker than maleic acid).

The third option is to increase the process economics of the dilute organic acid pretreatment by moving to another raw material altogether, in order to increase the total valorization. When a feedstock contains protein as well as lignocellulose, this protein may be hydrolyzed with a mild acid treatment and separated in order to create more value. This may be done by the application of the protein rich stream to animal feed or, potentially even more economically attractive, it can be used in the production of chemicals. The so called 'green chemistry' can use these streams as raw material for the production of nitrogen-containing chemicals from amino acids [59-61]. After protein extraction, the remaining lignocellulose fraction can be treated to yield fermentable sugars. Although the raw material might be more expensive, and the total process would be more complex, the total potential for valorization is also greater. In short, this implies the application of a more detailed biorefinery concept.

Something to keep in mind is that the production of second generation bioethanol can have several goals/reasons. For example, if first generation bioethanol production grows, as it is expected to do in the USA [62,63], large quantities of lignocellulose will become available. The current process can make use of that by-product stream, especially in areas where it is not economical to transport the straw over long distances. In short, in an area with a lot of first generation ethanol plants, lignocellulosic material such as wheat straw or corn stover may become very cheap and easily available. Moreover, in order to make first generation bioethanol more sustainable, it can be considered essential to use the lignocellulosic byproduct streams for second generation bioethanol production. Speaking in terms of carbon dioxide emissions, the additional (second generation) bioethanol would certainly speed up paying off carbon debts resulting from land use change in first generation bioethanol production [63,64].

A final point of interest is the application of a high solids loading in the pretreatment. In the current study, 10% (w/w) solids loading is applied. In previous work, it has been shown that raising the solids loading from 10 to 30% (w/w) does not negatively affect the pretreatment efficiency, as long as the acid concentration is raised accordingly [25]. However, combining these findings with the current study, a higher maleic acid concentration would result in higher acid costs. This is partly due to more maleic acid being isomerized or degraded, as well as to the fact that a relatively smaller fraction of the total liquid phase can be pressed out. For example, 30% (w/w) solids loading implies that only about a third of the total liquid phase can be pressed out in the solid-liquid separation (to 40% w/w dry matter). Increasing the solids loading, on the other hand, would lead to a lower processing cost per Mg of raw material. Obviously, also considering the effects on xylose concentration and furfural formation, there is a trade off concerning the pros and cons when raising the solids loading.

This study uses an extensive, but not exhaustive number of factors that influence the optimization of the maleic acid pretreatment of wheat straw. Since the focus is on the maleic acid pretreatment alone, and not on the whole integrated conversion process of wheat straw to bioethanol, some factors are not taken into account. These include factors like capital costs, enzyme costs, recycle costs and the possible benefits from the organic by-product stream that results from a conversion process with a maleic acid pretreatment.

## Conclusions

It is shown that the optimal process conditions in this study for the dilute maleic acid pretreatment of wheat straw are 50 min at 170°C with a maleic acid concentration of 46 mM. These conditions resulted in a theoretical optimal yield of 65.26 €/Mg dry straw, consisting of 68.03 € glucose benefits, 16.74 € xylose benefits, 16.55 € maleic acid costs, 2.06 € heating costs and 0.68 € NaOH costs. The most important cost factor in this study is the cost of replenishing maleic acid. In all but the most severe of the studied conditions, furfural formation was so limited that the associated costs are considered negligible. At the economic optimum, the total glucose yield from the pretreatment solids is 85% of the glucan originally present in the wheat straw. Xylose yield from the liquid phase after pretreatment is close to 80% of all xylan present. After this pretreatment, 7% to 10% of the glucose is expected to be present in monomeric and oligomeric form in the liquid phase. Nearly all xylose present in the liquid phase is in a monomeric form. Using the dilute maleic acid pretreatment and subsequent enzymatic hydrolysis, almost complete conversion of wheat straw glucan and xylan is possible and very high yields (approximately 90%-95%) can be achieved.

## Abbreviations

Mg: megagram (metric ton); GL: gigalitre; ha: hectare; HMF: 5-hydroxymethylfurfural; HPLC: high-performance liquid chromatography; Tg: teragram (million metric tons).

## Competing interests

This study was partly funded by CCL Research (Veghel, The Netherlands). AMJK and JPMS are mentioned as co-inventors on an indirectly related patent application, No. PCT/NL000125. The authors declare that they have no financial or non-financial competing interests.

## Authors' contributions

AMJK conceived of and designed the study, carried out the experiments, as well as the chemical and statistical analyses, and drafted the manuscript. HHB participated in the design of the study. HHB, ELS and JPMS helped to draft the manuscript. All authors read and approved the final manuscript.

## Supplementary Material

Additional file 1 **Statistics**. File with information on statistical analysis, ANOVA's and *R*^2^, of all response factors. Click here for file

## References

[B1] Food and Agriculture Organization of the United Nations Wheat and Maize Production Quantities EU 27 in 2007 2009 FAOSTAT

[B2] Kim S Dale BE Global potential bioethanol production from wasted crops and crop residues Biomass Bioenergy 2004 26 361 375 10.1016/j.biombioe.2003.08.002

[B3] US Department of Energy Theoretical Ethanol Yield Calculator http://www1.eere.energy.gov/biomass/ethanol_yield_calculator.html

[B4] US Department of Energy Biomass Feedstock Composition and Property Database. US-DOE 2009

[B5] Energy Information Administration Motor Gasoline Consumption in EU-27, from 2004 to 2007. EIA

[B6] US Department of Energy Alternative Fuels & Advanced Vehicle Data Centre, fuel properties http://www.afdc.energy.gov/afdc/fuels/properties.html

[B7] Yang B Wyman CE Pretreatment: the key to unlocking low-cost cellulosic ethanol Biofuels, Bioprod Bioref 2008 2 26 40 10.1002/bbb.49

[B8] Foust T Aden A Dutta A Phillips S An economic and environmental comparison of a biochemical and a thermochemical lignocellulosic ethanol conversion processes Cellulose 2009 16 547 565 10.1007/s10570-009-9317-x

[B9] Mosier N Wyman C Dale B Elander R Lee YY Holtzapple M Ladisch M Features of promising technologies for pretreatment of lignocellulosic biomass Bioresour Technol 2005 96 673 686 10.1016/j.biortech.2004.06.025 15588770

[B10] Lawford HG Rousseau JD Cellulosic fuel ethanol: alternative fermentation process designs with wild-type and recombinant *Zymomonas mobilis* Appl Biochem Biotechnol 2003 106 457 469 10.1385/ABAB:106:1-3:457 12721468

[B11] Wyman CE Dale BE Elander RT Holtzapple M Ladisch MR Lee YY Coordinated development of leading biomass pretreatment technologies Bioresour Technol 2005 96 1959 1966 10.1016/j.biortech.2005.01.010 16112483

[B12] Lloyd TA Wyman CE Combined sugar yields for dilute sulfuric acid pretreatment of corn stover followed by enzymatic hydrolysis of the remaining solids Bioresour Technol 2005 96 1967 1977 10.1016/j.biortech.2005.01.011 16112484

[B13] Zhu Z Sathitsuksanoh N Vinzant T Schell DJ McMillan JD Zhang YHP Comparative study of corn stover pretreated by dilute acid and cellulose solvent-based lignocellulose fractionation: Enzymatic hydrolysis, supramolecular structure and substrate accessibility Biotechnol Bioeng 2009 DOI: 10.1002/bit.22307 19337984 10.1002/bit.22307

[B14] Dunlop AP Furfural formation and behavior Ind Eng Chem 1948 40 204 209 10.1021/ie50458a006

[B15] McKibbins SW Harris JF Saeman JF Neill WK Kinetics of the acid catalyzed conversion of glucose to 5-hydroxymethyl-2-furaldehyde and levulinic acid Forest Prod J 1962 5 17 23

[B16] Qian XH Nimlos MR Davis M Johnson DK Himmel ME *Ab initio *molecular dynamics simulations of beta-D-glucose and beta-D-xylose degradation mechanisms in acidic aqueous solution Carbohydr Res 2005 340 2319 2327 10.1016/j.carres.2005.07.021 16095579

[B17] Palmqvist E Hahn-Hagerdal B Fermentation of lignocellulosic hydrolysates. II: inhibitors and mechanisms of inhibition Bioresour Technol 2000 74 25 33 10.1016/S0960-8524(99)00161-3

[B18] Cantarella M Cantarella L Gallifuoco A Spera A Alfani F Effect of inhibitors released during steam-explosion treatment of poplar wood on subsequent enzymatic hydrolysis and SSF Biotechnol Prog 2004 20 200 206 10.1021/bp0257978 14763843

[B19] Klinke HB Thomsen AB Ahring BK Inhibition of ethanol-producing yeast and bacteria by degradation products produced during pre-treatment of biomass Appl Microbiol Biotechnol 2004 66 10 26 10.1007/s00253-004-1642-2 15300416

[B20] Mosier NS Ladisch CM Ladisch MR Characterization of acid catalytic domains for cellulose hydrolysis and glucose degradation Biotechnol Bioeng 2002 79 610 618 10.1002/bit.10316 12209808

[B21] Lu Y Mosier NS Biomimetic catalysis for hemicellulose hydrolysis in corn stover Biotechnol Prog 2007 23 116 123 10.1021/bp060223e 17269678

[B22] Antal MJ Mok WSL Richards GN Kinetic studies of the reactions of ketoses and aldoses in water at high temperature. 1. Mechanism of formation of 5-(hydroxymethyl)-2-furaldehyde from D-fructose and sucrose Carbohydr Res 1990 199 91 109 10.1016/0008-6215(90)84096-D 2379202

[B23] Antal MJ Leesomboon T Mok WS Richards GN Kinetic studies of the reactions of ketoses and aldoses in water at high temperature. 3. Mechanism of formation of 2-furaldehyde from D-xylose Carbohydr Res 1991 217 71 85 10.1016/0008-6215(91)84118-X

[B24] Mosier NS Sarikaya A Ladisch CM Ladisch MR Characterization of dicarboxylic acids for cellulose hydrolysis Biotechnol Prog 2001 17 474 480 10.1021/bp010028u 11386868

[B25] Kootstra AMJ Beeftink HH Scott EL Sanders JPM Comparison of dilute mineral and organic acid pretreatment for enzymatic hydrolysis of wheat straw Biochem Eng J 2009 46 126 131 10.1016/j.bej.2009.04.020

[B26] Partanen KH Mroz Z Organic acids for performance enhancement in pigs Nutr Res Rev 1999 12 117 145 10.1079/095442299108728884 19087448

[B27] Radecki SV Juhl MR Miller ER Fumaric and citric acids as feed additives in starter pig diets: effect on performance and nutrient balance J Anim Sci 1988 66 2598 2605 3198539 10.2527/jas1988.66102598x

[B28] Pérez JA Effect of process variables on liquid hot water pretreatment of wheat straw for bioconversion to fuel-ethanol in a batch reactor J Chem Technol Biotechnol 2007 82 929 938 10.1002/jctb.1765

[B29] García-Cubero MT González-Benito G Indacoechea I Coca M Bolado S Effect of ozonolysis pretreatment on enzymatic digestibility of wheat and rye straw Bioresour Technol 2009 100 1608 1613 10.1016/j.biortech.2008.09.012 18951781

[B30] Lu X Zhang Y Angelidaki I Optimization of H2SO4-catalyzed hydrothermal pretreatment of rapeseed straw for bioconversion to ethanol: Focusing on pretreatment at high solids content Bioresour Technol 2009 100 3048 3053 10.1016/j.biortech.2009.01.008 19268577

[B31] Petersen MØ Larsen J Thomsen MH Optimization of hydrothermal pretreatment of wheat straw for production of bioethanol at low water consumption without addition of chemicals Biomass Bioenergy 2009 33 834 840 10.1016/j.biombioe.2009.01.004

[B32] Technical Association of the Pulp and Paper Industry T 412 om-02; Moisture in Pulp, Paper and Paperboard 2004 TAPPI, Georgia

[B33] Technical Association of the Pulp and Paper Industry T 204 cm-97; Solvent Extractives of Wood and Pulp 2004 TAPPI, Georgia

[B34] Technical Association of the Pulp and Paper Industry T 249 cm-00; Carbohydrate Composition of Extractive-free Wood and Wood Pulp by Gas-liquid Chromatography 2004 TAPPI, Georgia

[B35] Technical Association of the Pulp and Paper Industry T 222 om-02; Acid-insoluble Lignin in Wood and Pulp 2004 TAPPI, Georgia

[B36] Technical Association of the Pulp and Paper Industry T 211 om-02; Ash in Wood, Pulp, Paper and Paperboard: Combustion at 525 °C 2004 TAPPI, Georgia

[B37] Technical Association of the Pulp and Paper Industry T 418 cm-97; Organic Nitrogen in Paper and Paperboard 2004 TAPPI, Georgia

[B38] Kabel MA Maarel MJEC van der Klip G Voragen AGJ Schols HA Standard assays do not predict the efficiency of commercial cellulase preparations towards plant materials Biotechnol Bioeng 2006 93 56 63 10.1002/bit.20685 16196058

[B39] Ruiz R Ehrman T Dilute Acid Hydrolysis Procedure for Determination of Total Sugars in Liquid Fractions of Process Samples. LAP-014 NREL analytical procedure 1996 Colorado: National Renewable Energy Laboratory

[B40] Sugar: World Production Supply and Distribution 2009 http://www.fas.usda.gov/htp/sugar/2009/May%20sugar%202009.pdf

[B41] ICIS-pricing: Maleic Anhydride Price; 6 February 2009 2009 Reed Business Information Ltd, Sutton, UK http://www.icispricing.com

[B42] Nozaki K Ogg R cis-trans isomerizations. I. The mechanism of a catalyzed isomerization of maleic acid to fumaric acid J Am Chem Soc 1941 63 2583 2586 10.1021/ja01855a013

[B43] Felthouse TR Burnett JC Horrell B Mummey MJ Kuo Y-J Maleic anhydride, maleic acid, and fumaric acid 2001 Kirk-Othmer Encyclopedia of Chemical Technology; Wiley

[B44] Weiss JM Preliminary study on the formation of malic acid J Am Chem Soc 1922 44 1118 10.1021/ja01426a025

[B45] Hojendahl K On isothermal reaction, velocity in homo-hetero-geneous systems in the absence of solvent: with special reference to the conversion of fused maleic acid into fumaric and malic acids J Phys Chem 1924 28 758 768 10.1021/j150241a007

[B46] ICIS-pricing: Caustic Soda Price; 6 February 2009 2009 Reed Business Information Ltd, Sutton, UK http://www.icispricing.com

[B47] Kootstra AMJ Mosier NS Scott EL Beeftink HH Sanders JPM Differential effects of mineral and organic acids on the kinetics of arabinose degradation under lignocellulose pretreatment conditions Biochem Eng J 2009 43 92 97 10.1016/j.bej.2008.09.004

[B48] Liu Z Slininger P Gorsich S Enhanced biotransformation of furfural and hydroxymethylfurfural by newly developed ethanologenic yeast strains Appl Biochem Biotechnol 2005 121 451 460 10.1385/ABAB:121:1-3:0451 15917621

[B49] Liu Z Genomic adaptation of ethanologenic yeast to biomass conversion inhibitors Appl Microbiol Biotechnol 2006 73 27 36 10.1007/s00253-006-0567-3 17028874

[B50] Almeida JRM Modig T Petersson A Hähn-Hägerdal B Lidén G Gorwa-Grauslund MF Increased tolerance and conversion of inhibitors in lignocellulosic hydrolysates by *Saccharomyces cerevisiae* J Chem Technol Biotechnol 2007 82 340 349 10.1002/jctb.1676

[B51] Heer D Sauer U Identification of furfural as a key toxin in lignocellulosic hydrolysates and evolution of a tolerant yeast strain Microb Biotechnol 2008 1 497 506 10.1111/j.1751-7915.2008.00050.x PMC3815291 21261870

[B52] Liu LZ Ma M Song M Evolutionarily engineered ethanologenic yeast detoxifies lignocellulosic biomass conversion inhibitors by reprogrammed pathways Mol Genet Genomics 2009 282 233 244 10.1007/s00438-009-0461-7 19517136 PMC3025311

[B53] Energy Information Administration Average Weekly Coal Commodity Spot Prices. EIA 2009

[B54] He F Yi W Bai X Investigation on caloric requirement of biomass pyrolysis using TG-DSC analyzer Energy Conversion Manag 2006 47 2461 2469 10.1016/j.enconman.2005.11.011

[B55] Eggeman T Elander RT Process and economic analysis of pretreatment technologies Bioresour Technol 2005 96 2019 2025 10.1016/j.biortech.2005.01.017 16112490

[B56] DACE Price booklet; Dutch Association of Cost Engineers 2009 27 Reed Business bv; M.M.M. Gianotten

[B57] Kabel MA Bos G Zeevalking J Voragen AGJ Schols HA Effect of pretreatment severity on xylan solubility and enzymatic breakdown of the remaining cellulose from wheat straw Bioresour Technol 2007 98 2034 2042 10.1016/j.biortech.2006.08.006 17029957

[B58] Maas R Bakker R Eggink G Weusthuis R Lactic acid production from xylose by the fungus Rhizopus oryzae Appl Microbiol Biotechnol 2006 72 861 868 10.1007/s00253-006-0379-5 16528511

[B59] Scott E Peter F Sanders J Biomass in the manufacture of industrial products--the use of proteins and amino acids Appl Microbiol Biotechnol 2007 75 751 762 10.1007/s00253-007-0932-x 17387469 PMC1914281

[B60] Konst PM Franssen MCR Scott EL Sanders JPM A study on the applicability of L-aspartate α-decarboxylase in the biobased production of nitrogen containing chemicals Green Chem 2009 11 1646 1652 10.1039/b902731a

[B61] Lammens TM De Biase D Franssen MCR Scott EL Sanders JPM The application of glutamic acid α-decarboxylase for the valorization of glutamic acid Green Chem 2009 11 1562 1567 10.1039/b913741f

[B62] Tokgoz S Elobeid A Fabiosa JF Hayes DJ Babcock BA Yu T-HE Dong F Hart CE Beghin JC Emerging biofuels: outlook of effects on U.S. grain, oilseed, and livestock markets Staff General Research Papers;12812 2007 Iowa State University: Iowa State University, Department of Economics

[B63] Searchinger T Heimlich R Houghton RA Dong F Elobeid A Fabiosa J Tokgoz S Hayes D Yu T-H Use of U.S. croplands for biofuels increases greenhouse gases through emissions from land-use change Science 2008 319 1238 1240 10.1126/science.1151861 18258860

[B64] Fargione J Hill J Tilman D Polasky S Hawthorne P Land clearing and the biofuel carbon debt Science 2008 319 1235 1238 10.1126/science.1152747 18258862

