# Supplementary material for: Optimization of the dilute maleic acid pretreatment of wheat straw
Source: Biotechnol Biofuels. 2009 Dec 21;2:31. doi: 10.1186/1754-6834-2-31 (PMC2806341; doi:10.1186/1754-6834-2-31)
Supplement: Additional file 1 — Statistics. File with information on statistical analysis, ANOVA's and R2, of all response factors. [file 1754-6834-2-31-S1.PDF]

Glucose benefits

Factor	Coefficient Estimate	df	Standard Error	95% CI Low	95% CI High
Intercept	53.1117	1	1.115815	50.70113	55.52227
Block 1	0.745003	2			
Block 2	-0.429903				
Block 3	-0.315099				
A-Pre time	4.028366	1	1.028159	1.807163	6.249569
B-Pre Tem	12.00357	1	1.028159	9.782372	14.22478
C-Maleic a	10.35827	1	1.028159	8.137071	12.57948
C^2	-9.830231	1	1.592817	-13.2713	-6.389159

ANOVA for Response Surface Reduced Quadratic Model

Analysis of variance table [Partial sum of squares - Type III]

Source	Sum of Squares	df	Mean Square	F Value	p-value Prob > F			
Block	67.156386	2	33.57819				0.957	R2
Model	3078.7129	4	769.6782	72.80959	< 0.0001	significant	0.944	R2 adj
A-Pre tir	162.27732	1	162.2773	15.35102	0.0018			
B-Pre Te	1440.858	1	1440.858	136.3015	< 0.0001			
C-Maleic	1072.9383	1	1072.938	101.4972	< 0.0001			
C^2	402.63934	1	402.6393	38.08865	< 0.0001			
Residual	137.42445	13	10.57111					
Lack of Fit	136.02462	10	13.60246	29.15179	0.0091	significant		
Pure Error	1.3998245	3	0.466608					
Cor Total	3283.2938	19						

Xylose benefits

Factor	Coefficient Estimate	df	Standard Error	95% CI Low	95% CI High
Intercept	14.31705	1	0.601537	13.02688	15.60722
Block 1	0.964818	2			
Block 2	-0.522899				
Block 3	-0.441919				
B-Pre Tem	2.974675	1	0.554282	1.785857	4.163492
C-Maleic a	5.132318	1	0.554282	3.943501	6.321135
C^2	-5.245643	1	0.85869	-7.08735	-3.403935

ANOVA for Response Surface Reduced Quadratic Model

Analysis of variance table [Partial sum of squares - Type III]

Source	Sum of Squares	df	Mean Square	F Value	p-value Prob > F			
Block	17.770819	2	8.885409				0.916	R^2
Model	466.54698	3	155.5157	50.61883	< 0.0001	significant	0.898	R^2 adj
B-Pre Te	88.486896	1	88.4869	28.80162	< 0.0001			
C-Maleic	263.40689	1	263.4069	85.73637	< 0.0001			
C^2	114.6532	1	114.6532	37.3185	< 0.0001			
Residual	43.012042	14	3.072289					
Lack of Fit	42.956074	11	3.905098	209.3223	0.0005	significant		
Pure Error	0.0559677	3	0.018656					
Cor Total	527.32984	19						

(Acid costs)^0.5

Factor	Coefficient Estimate	df	Standard Error	95% CI Low	95% CI High
Intercept	2.872143	1	0.034905	2.793184	2.951103
Block 1	-0.163568	2			
Block 2	0.247284				
Block 3	-0.083716				
A-Pre time	0.227573	1	0.029618	0.160574	0.294573
B-Pre Tem	0.560977	1	0.029618	0.493977	0.627977
C-Maleic a	1.575389	1	0.029618	1.508389	1.642389
AB	0.245813	1	0.033113	0.170905	0.32072
AC	0.119687	1	0.033113	0.044779	0.194595
BC	0.382614	1	0.033113	0.307706	0.457522
B^2	0.370056	1	0.053717	0.248539	0.491573
C^2	-0.448037	1	0.053717	-0.569554	-0.32652

ANOVA for Response Surface Reduced Quadratic Model

Analysis of variance table [Partial sum of squares - Type III]

Source	Sum of Squares	df	Mean Square	F Value	p-value Prob > F			
Block	0.548	2.000	0.273993				0.997	R^2
Model	30.941	8.000	3.867628	440.905	< 0.0001	significant	0.995	R^2 adj
A-Pre tim	0.518	1.000	0.517896	59.03949	< 0.0001			
B-Pre Temper	3.147	1.000	3.146953	358.7489	< 0.0001			
C-Maleic acid	24.819	1.000	24.8185	2829.28	< 0.0001			
AB	0.483	1.000	0.483391	55.10597	< 0.0001			
AC	0.115	1.000	0.1146	13.06429	0.0056			
BC	1.171	1.000	1.171148	133.5095	< 0.0001			
B^2	0.416	1.000	0.416302	47.4579	< 0.0001			
C^2	0.610	1.000	0.610241	69.56676	< 0.0001			
Residual	0.079	9.000	0.008772					
Lack of Fit	0.0636075	6	0.010601	2.07317	0.2934	not significant		
Pure Error	0.0153406	3	0.005114					
Cor Total	31.567956	19						

NaOH costs

Factor	Coefficient Estimate	df	Standard Error	95% CI Low	95% CI High
Intercept	0.982634	1	0.020664	0.938828	1.02644
Block 1	0.009952	2			
Block 2	0.002787				
Block 3	-0.012739				
C-Maleic a	0.825155	1	0.028957	0.763769	0.886541

ANOVA for Response Surface Reduced Linear Model

Analysis of variance table [Partial sum of squares - Type III]

Source	Sum of Squares	df	Mean Square	F Value	p-value Prob > F			
							0.981	R^2
Block	0.0019066	2	0.000953				0.979	R^2 adj
Model	6.8088067	1	6.808807	812.025	< 0.0001	significant		
C-Maleic	6.8088067	1	6.808807	812.025	< 0.0001			
Residual	0.1341596	16	0.008385					
Lack of Fit	0.1207683	13	0.00929	2.081176	0.2986	not significant		
Pure Error	0.0133913	3	0.004464					
Cor Total	6.9448729	19						

Log10(Furfural concentration)

Factor	Coefficient Estimate	df	Standard Error	95% CI Low	95% CI High
Intercept	-0.421494	1	0.046107	-0.521953	-0.321035
Block 1	0.042894	2			
Block 2	-0.009522				
Block 3	-0.033372				
A-Pre time	0.213288	1	0.042485	0.12072	0.305855
B-Pre Tem	0.566968	1	0.042485	0.474401	0.659536
C-Maleic a	0.328423	1	0.042485	0.235856	0.42099
BC	0.166457	1	0.0475	0.062964	0.26995
C^2	-0.22436	1	0.065818	-0.367764	-0.080955

ANOVA for Response Surface Reduced Quadratic Model

Analysis of variance table [Partial sum of squares - Type III]

Source	Sum of Squares	df	Mean Square	F Value	p-value Prob > F			
							0.960	R^2
Block	0.0172938	2	0.008647				0.943	R^2 adj
Model	5.1794656	5	1.035893	57.39058	< 0.0001	significant		
A-Pre tir	0.4549159	1	0.454916	25.20326	0.0003			
B-Pre Te	3.214532	1	3.214532	178.0916	< 0.0001			
C-Maleic	1.0786159	1	1.078616	59.75751	< 0.0001			
BC	0.2216629	1	0.221663	12.28057	0.0043			
C^2	0.2097389	1	0.209739	11.61996	0.0052			
Residual	0.2165986	12	0.01805					
Lack of Fit	0.2157627	9	0.023974	86.04659	0.0018	significant		
Pure Error	0.0008358	3	0.000279					
Cor Total	5.413358	19						

Heating costs

Factor	Coefficient Estimate	df	Standard Error	95% CI Low	95% CI High
Intercept	1.784617	1	3.48E-07	1.784616	1.784617
Block 1	-2.74E-07	2			
Block 2	7.83E-07				
Block 3	-5.09E-07				
B-Pre Tem	0.274556	1	4.88E-07	0.274555	0.274557

ANOVA for Response Surface Reduced Linear Model

Analysis of variance table [Partial sum of squares - Type III]

Source	Sum of Squares	df	Mean Square	F Value	p-value Prob > F			
							1.000	R^2
Block	0	2	0				1.000	R^2 adj
Model	0.7538101	1	0.75381	63660000	< 0.0001	significant		
B-Pre Te	0.7538101	1	0.75381	63660000	< 0.0001			
Residual	0	16	0					
Lack of Fit	0	13	0					
Pure Error	0	3	0					
Cor Total	0.7538101	19						
